# Attitudes of people with multiple sclerosis toward brain donation

**DOI:** 10.3389/fneur.2023.1115303

**Published:** 2023-01-26

**Authors:** Ruth Ann Marrie, Leanne Kosowan, Gary R. Cutter, Robert J. Fox, Amber Salter

**Affiliations:** ^1^Department of Internal Medicine, Rady Faculty of Health Sciences, Max Rady College of Medicine, University of Manitoba, Winnipeg, MB, Canada; ^2^Department of Community Health Sciences, Rady Faculty of Health Sciences, Max Rady College of Medicine, University of Manitoba, Winnipeg, MB, Canada; ^3^Department of Family Medicine, Rady Faculty of Health Sciences, Max Rady College of Medicine, University of Manitoba, Winnipeg, MB, Canada; ^4^Department of Biostatistics, University of Alabama in Birmingham, Birmingham, AL, United States; ^5^Mellen Center for Multiple Sclerosis, Neurological Institute, Cleveland Clinic, Cleveland, OH, United States; ^6^Department of Neurology, UT Southwestern, Dallas, TX, United States

**Keywords:** multiple sclerosis, brain donation, attitudes, survey, cross-sectional study

## Abstract

**Objective:**

Research directly examining brain tissue has played an important role in understanding the pathology and pathogenesis of multiple sclerosis (MS) and other diseases of the central nervous system. Such research relies heavily on donations of post-mortem brain tissue yet little is known about the attitudes of people with multiple sclerosis (MS) about brain donation. We aimed to assess the attitudes of people with MS toward brain donation, their preferences related to discussions of brain donation, and factors associated with attitudes toward brain donation including sociodemographic and clinical characteristics, health literacy and religiosity.

**Methods:**

In a cross-sectional study, we surveyed participants in the North American Research Committee on Multiple Sclerosis (NARCOMS) Registry regarding their attitudes toward brain donation, reasons for participating or not participating in brain donation, and related communication preferences. We used multivariable logistic regression analyses to test factors associated with attitudes regarding brain donation.

**Results:**

Most of the 4,520 participants were women (80.8%), self-identified as white (88.1%), with a post-secondary education, functional health literacy and moderate-severe disability. Sixty-two percent of participants would consider brain donation. Factors associated with considering brain donation included female gender, having a post-secondary education, being physically active, having moderate-severe disability and more comorbidities, and alcohol intake. Seventy-five percent of participants indicated that they preferred to receive information regarding brain donations from physicians.

**Conclusion:**

Two-thirds of people with MS would consider brain donation. People with MS desire to hear about brain donation from their health care providers rather than other sources.

## 1. Introduction

Research directly examining brain tissue has played an important role in understanding the pathology and pathogenesis of multiple sclerosis (MS) and other diseases of the central nervous system. Such research relies heavily on donations of post-mortem brain tissue because autopsy rates continue to decline and typical post-mortem intervals for autopsy are too long for most research techniques (1). A recent initiative of the European Charcot Foundation highlighted the importance of tissue donations including post-mortem brain tissue, for MS research and the need to increase the availability of brain tissue, particularly from patients with early MS or cause of death unrelated to MS (2). Specifically, the initiative pointed to unresolved questions regarding the mechanisms underlying brain and spinal cord injury, such as the initial trigger of inflammation, or whether infectious agents play a mechanistic role in the disease process. Presently, most post-mortem brain tissue is obtained from individuals with a long disease duration. The initiative recommended increasing the availability of brain tissue from people with early MS an aggressive disease course or those who had a cause of death unrelated to MS. The value of obtaining post-mortem brain tissue from other neuroinflammatory and neurodegenerative diseases for comparative purposes was also emphasized. A more comprehensive understanding of the pathogenesis of the disease offers the opportunity for development of more effective interventions. Increasing the number of brain donations requires an understanding of barriers and facilitators, including attitudes toward donation. Such attitudes may vary across patient populations.

Relatively little is known about the attitudes of people with MS regarding brain donation. A 2019 systematic review of qualitative or mixed methods studies regarding attitudes, motivations and feelings about brain donation did not include any studies involving people with MS (3). A cross-sectional study of participants with neurodegenerative and non-neurodegenerative disorders grouped together participants with MS, stroke, epilepsy, headache disorders and other demyelinating disorders; the number of participants with MS was not reported explicitly. That study found that about half of participants would consider brain donation (4). Factors that positively influenced brain donation included altruism, religious motivation, while factors that negatively influenced brain donation included fears of being disfigured and perceived stress for family members (4). The potential role of health literacy was not evaluated despite the importance of health literacy in effective communication, and understanding and processing of complex health-related information (5, 6).

We aimed to assess the attitudes of people with MS toward brain donation, their preferences related to discussions of brain donation, and factors associated with attitudes toward brain donation including sociodemographic and clinical characteristics, health literacy and religiosity.

## 2. Methods

### 2.1. Study population

We conducted this cross-sectional study using the North American Research Committee on Multiple Sclerosis Registry (NARCOMS), a self-report registry for persons with MS (7). Participants in the registry complete questionnaires at enrollment, and update their demographic and clinical information and respond to questions regarding special topics of interest *via* semi-annual surveys. Questionnaires are completed on paper or online, according to participant preference. Participants permit use of their de-identified information for research purposes. At the time of this survey, the NARCOMS registry was approved by the Institutional Review Board at UT Southwestern.

### 2.2. Attitudes toward brain donation

In the fall of 2021 we surveyed participants regarding brain donation. According to a 2018 systematic review the brain donation decision involves contextual knowledge (knowledge of donations and donation process, health literacy), conceptual understanding (altruism, religious, spiritual), personal experience, time and process (communication with professionals, healthcare experience, timing of donation request), and family/friends matter (family's opinion and discussion within the family) (3). Therefore, we surveyed participants regarding their attitudes toward brain donation, the reasons they would or would not participate, and their preferences related to discussions of brain donation including the preferred person initiating those discussions, and timing of the discussion. As described further below, we also captured information regarding religiosity and health literacy.

This section of the survey provided participants with the following explanation regarding brain donation: “Brain donation is when a person and their family decide to donate their brain for medical research after their death. Brain donation helps to improve the understanding of diseases like MS that affect the brain. Signing up to be an organ donor does not include donating the brain.” We employed questions from a study conducted in participants with neurodegenerative and non-neurodegenerative disorders (4). We asked participants to evaluate five statements: (i) I would not feel offended if I were asked to participate in a brain donation; (ii) I am aware of the importance of brain donation for research; (iii) I believe research staff will handle donated brain samples properly and professionally; (iv) I would consider donating my brain for research; and (v) I would be agreeable to allow my family to make the decision whether or not to donate my brain for research. Responses for each statement used a 5-point Likert-type scale with responses including strongly disagree, disagree, neutral/not sure, agree and strongly agree.

Participants selected all statements that reflected the most applicable reasons that would affect their decision to participate in brain donation for research including: (i) the donation could benefit other patients in the future; (ii) the donation will give closure to my family; (iii) the donation will advance medical knowledge on my condition; (iv) the donation will help my family recognize the potential heritability of my disorder; and (v) other (specify). Similarly, participants selected all statements that reflected the most applicable reasons that would affect their decision not to participate in brain donation including: (i) the donation procedure will disfigure my body; (ii) the donation procedure is against my religious beliefs; (iii) I need more time to think about it; (iv) process too troublesome; (v) conservative mindset; (vi) unwilling for body to go through any more procedures; (vii) the donation procedure will be too stressful for my family members; (viii) the donation procedure will delay funeral rites and arrangements; (ix) I am skeptical about research and its benefits; (x) I need more information about brain donation; (xi) no reasons; and (xii) other (specify).

Participants reported their communication preferences regarding the discussion of brain donation using three questions. They indicated the most preferred person to discuss brain donation where response options were: your treating neurologist, any other doctor, brain donation coordinator, nurse, research staff or others (specify). Participants reported the most preferred way to receive information regarding brain donation, where response options were: doctors, family members, friends, patient support group meetings, new media (e.g., social media), traditional media (e.g., television), and others (specify). Finally, participants reported the most appropriate time for medical staff to approach them regarding brain donation options, where response options were: first clinic visit, any time after the first clinic visit, never, not sure, and others (specify).

### 2.3. Covariates

From the enrollment questionnaire, we obtained information regarding participant characteristics of gender (male as reference), date of birth, race and ethnicity, education level, and age of symptom onset (from which we derived disease duration). From the fall 2021 questionnaire, we obtained information regarding annual household income, comorbidities, health behaviors, disability status, current clinical course, religiosity and health literacy. Based on the distribution of responses we categorized race as white (reference) vs. other, and education as high school/General Educational Development Test (reference), and post-secondary (Associate's Degree, Bachelor's Degree, Post-graduate education, and Technical degree). Annual household income was categorized as < $50,000 [reference], $50,001–100,000, >$100,000 and “I do not wish to answer.” Participants reported whether a doctor had diagnosed them with comorbidities including anxiety disorder, depression, autoimmune thyroid disease, diabetes, hypertension, hyperlipidemia, heart disease, chronic lung disease, irritable bowel syndrome, psoriasis, fibromyalgia, sleep apnea, stroke and cancer; these were aggregated as a count [0 (reference), 1, 2, ≥3]. Participants reported current smoking status [yes vs. no (reference)], any physical activity [yes vs. no (reference)], and alcohol intake (never, monthly or less, two to four times a month, two to three times a week, four or more times a week). We dichotomized alcohol intake as no (reference group) vs. yes. Disability status was measured using the Patient Determined Disease Steps, a single item measure with eight potential responses ranging from 0 (normal) to 8 (bedridden). This self-report measure correlates strongly with the physician-scored Expanded Disability Status Scale. We categorized the PDDS as mild (0–1), moderate (2–4) and severe (5–8) (8). Clinical course was categorized as clinically isolated syndrome, relapsing remitting, primary/secondary progressive (reference group) and don't know.

We assessed religiosity using the Duke University Religion Index (DUREL), a measure developed for use in epidemiologic studies, (9) and used in a prior study of physician-assisted dying in the NARCOMS population (8). The DUREL includes five items that assess organizational religious activity, non-organizational religious activity and subjective religiosity, and has high internal consistency reliability, high test-retest reliability, and convergent validity with other measures of religiosity. Total scores range from 5 to 27, where higher scores indicate higher religiosity. We categorized religiosity as 5–10 (low religiosity; reference group), 11–16, 17–22, and 23–27 (high religiosity), consistent with our prior work (8). Although the DUREL can generate three subscale scores, we did not include them in the models because the authors of the DUREL advise against this due to possible collinearity between the subscales or because subscale scores may cancel each other out (9). We did not query specific religions because our prior experience using the DUREL indicated that this is a highly sensitive issue for NARCOMS participants.

We assessed health literacy using the Medical Term Recognition Test (METER), a brief, self-administered questionnaire comprised of 40 medical words and 30 non-words (10). The respondents indicates the words they recognize as actual medical words, and the number of incorrectly identified words is subtracted from the correctly identified words to generate a total score. Scores of 0–20 indicate low literacy, 21–34 indicate marginal literacy and 35–40 indicate functional literacy; functional literacy was used as the reference group in regression analyses.

### 2.4. Statistical analysis

We characterized the study population using descriptive statistics including mean [standard deviation (SD)], median [interquartile range (IQR)] and frequency (percent). Similarly, we used descriptive statistics to summarize attitudes toward brain donation, communication preferences and reasons for agreeing to or declining brain donation participation. For bivariate analyses, we used student's *t*-tests, Wilcoxon or Kruskal-Wallis tests and chi-square tests, as appropriate.

For each of the five attitude statements regarding brain donation, we examined factors associated with choosing agree/strongly agree (“agree”) as compared to all other responses (“disagree”) using binary logistic regression. Each model included gender, age, education, income, comorbidities, smoking status, physical activity, alcohol intake, disability status, clinical course, disease duration, religiosity, and health literacy, as defined above. We tested for an interaction between clinical course and PDDS group using a multiplicative interaction term and a likelihood ratio test assessing whether all two-way interactions were equal to zero. Model assumptions were assessed using standard methods. Model fit was assessed using the Hosmer Lemeshow Goodness of Fit statistic, and we report model c-statistics.

Statistical analyses were conducted using SAS V9.4 (SAS Institute Inc., Cary, NC).

## 3. Results

The survey was distributed to 8,568 NARCOMS participants, of whom 5,719 (66.7%) responded. As compared to non-responders, responders were more likely to be white, had a higher level of education, were slightly younger at enrollment and had less disability at enrollment although most of these differences were too small to be clinically meaningful (Supplementary Table 1). After application of the inclusion criteria, 4,520 participants constituted the final sample. Most participants were women, white, with a post-secondary education, functional health literacy and a moderate to severe level of disability (Table 1). Over three-quarters of participants had a physical comorbidity (*n* = 3,738), the most common of which were hyperlipidemia (*n* = 1,675, 37.1%), hypertension (*n* = 1,670, 37.0%), depression (*n* = 1,640, 36.3%), osteoporosis (*n* = 1,051, 23.9%) and anxiety (*n* = 847, 18.7%).

**Table 1:** Characteristics of study participants (*n* = 4,520).

**Characteristics**	**Value**
Age at time of fall 2021 survey (years), mean (SD)	63.2 (10.1)
Age at MS symptom onset (years), mean (SD)	31.9 (9.6)
Age at MS diagnosis (years), mean (SD)	39.1 (9.8)
Disease duration (years), mean (SD)	31.2 (11.5)
Clinical course, *n* (%)	
Clinically isolated syndrome	67 (1.5)
Relapsing remitting	2,412 (53.7)
Primary or secondary progressive	1,605 (35.7)
Don't know	408 (9.1)
Female, *n* (%)	3,652 (80.8)
White race, *n* (%)	3,984 (88.1)
Education at enrollment, *n* (%)^a^	
≤ High school/GED	1,030 (23.7)
≥Post-secondary	3,320 (76.3)
Annual household income, *n* (%)^b^	
< $50,000	1,378 (30.6)
$50,001–100,000	1,125 (25.0)
Over $100,000	1,020 (22.7)
I do not wish to answer	974 (21.7)
PDDS, *n* (%)^c^	
Mild (0–1)	1,421 (32.0)
Moderate (2–4)	1,435 (32.3)
Severe (5–8)	1,585 (35.7)
Number of comorbidities, *n* (%)	
0	998 (22.1)
1	1,219 (27.0)
2	1,009 (22.3)
≥3	1,294 (28.6)
Current smoking status, *n* (%)^d^	
Yes	276 (6.1)
No	4,230 (93.9)
Alcohol intake, *n* (%)	
Never	1,553 (34.4)
Monthly or less	1,164 (25.8)
Two to four times per month	725 (16.1)
Two to three times per week	488 (10.8)
Four or more times per week	584 (12.9)
Any physical activity, *n* (%)^e^	2,659 (65.6)
Religiosity, *n* (%)^f^	
5–10	1,314 (29.7)
11–16	555 (12.3)
17–22	431 (9.5)
23–27	2,193 (48.5)
Health literacy, *n* (%)	
Low	77 (1.7)
Marginal	673 (15.1)
Functional	3,716 (83.2)

Data missing for some respondents (n = ) ^a^170, ^b^23, ^c^79, ^d^14, ^e^6, ^f^19.

PDDS, Patient Determined Disease Steps.

### 3.1. Attitudes toward brain donation

**Figure 1:**
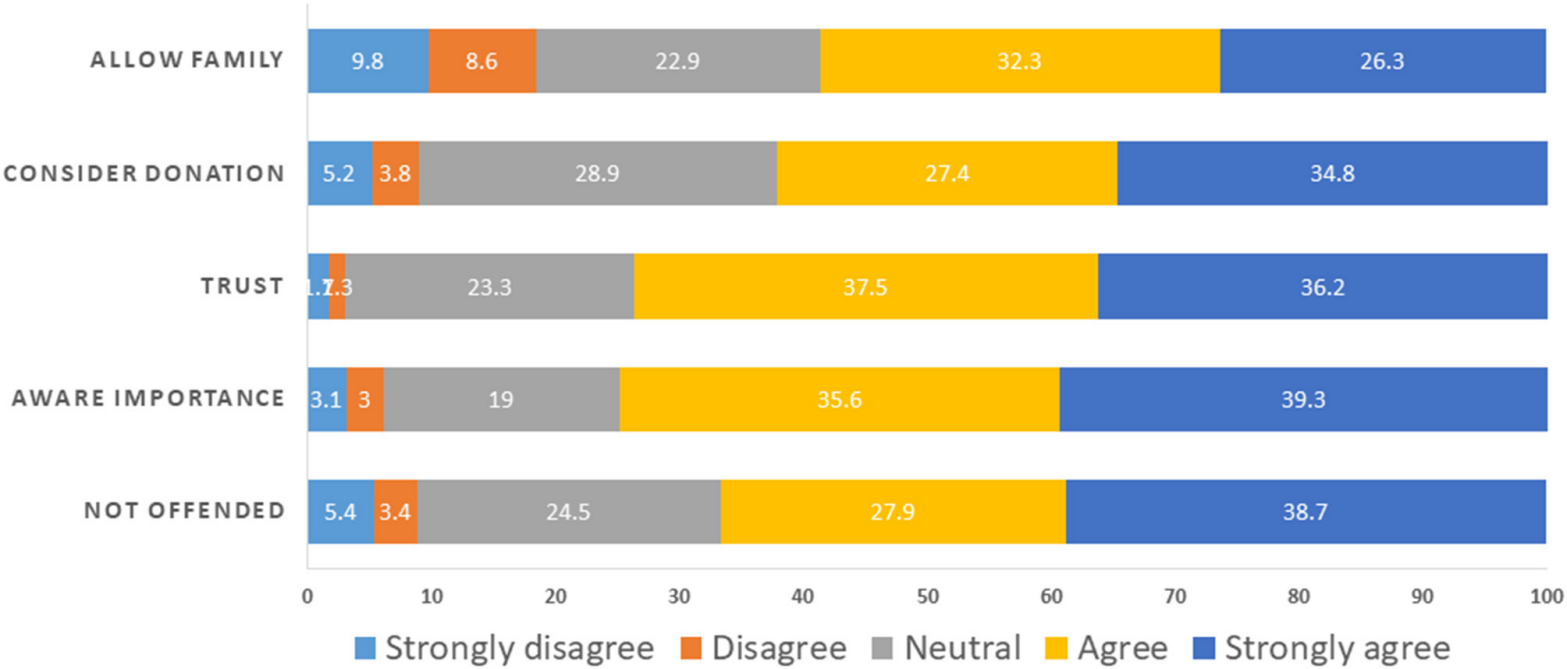
Attitudes toward brain donation. Participants evaluated five statements: (i) I would not feel offended if I were asked to participate in a brain donation = NOT OFFENDED; (ii) I am aware of the importance of brain donation for research = AWARE IMPORTANCE; (iii) I believe research staff will handle donated brain samples properly and professionally = TRUST; (iv) I would consider donating my brain for research = CONSIDER DONATION; and (v) I would be agreeable to allow my family to make the decision whether or not to donate my brain for research = ALLOW FAMILY.

When we asked participants about their agreement with five attitude statements regarding brain donation (I would not feel offended if I were asked to participate in a brain donation; I am aware of the importance of brain donation for research; I believe research staff will handle donated brain samples properly and professionally; I would consider donating my brain for research; and I would be agreeable to allow my family to make the decision whether or not to donate my brain for research), over half of respondents agreed or strongly agreed with the five statements (Figure 1). Agreement was lowest (58.6%) for being agreeable to allow family to make the decision, and it was highest for awareness of the importance of brain donation, and trust that the brain samples would be handled appropriately. Nineteen to twenty-nine percent of respondents indicated that they were neutral or unsure for these statements.

On multivariable logistic regression analyses several factors were associated with attitudes toward brain donation (Table 2). Factors associated with agreement with the statement that they would not be offended if asked to participate in a brain donation included post-secondary education as compared to less than post-secondary education, and being physical active (vs. inactive). Factors associated with disagreeing with the statement included being a current smoker, having a higher degree of religiosity, and having marginal or low, rather than functional health literacy. We did not observe an interaction between clinical course and disability (X^2^ = 8.84, *p* = 0.18).

**Table 2:** Odds ratios (95% confidence intervals) for factors associated with attitudes toward brain donation.

**Characteristics**	**Not** **offended**	**Aware** **important**	**Trust** **staff**	**Consider** **donation**	**Agree** **family**
Age	0.99 (0.98–1.0)	1.0 (0.99–1.01)	0.99 (0.98–1.0)	**0.98** **(0.97–0.99)**	0.99 (0.99–1.0)
Disease duration	1.0 (0.99–1.01)	**1.01** **(1.0–1.01)**	1.0 (0.99–1.01)	**1.01** **(1.0–1.01)**	**1.0** **(1.0–1.01)**
Female	1.05 (0.88–1.25)	**1.45** **(1.21–1.74)**	1.01 (0.84–1.22)	**1.25** **(1.05–1.48)**	1.0 (0.85–1.18)
Race					
White	1.0	1.0	1.0	1.0	1.0
Other	0.86 (0.7–1.05)	0.90 (0.73–1.12)	0.89 (0.72–1.10)	1.0 (0.82–1.23)	0.98 (0.81–1.19)
Education at enrollment					
≤ High school/ GED	1.0	1.0	1.0	1.0	1.0
≥Post-secondary	**1.41** **(1.20–1.65)**	**1.28** **(1.08–1.51)**	**1.29** **(1.09–1.52)**	**1.48** **(1.27–1.73)**	**1.29** **(1.11–1.50)**
Annual household income					
< $50,000	1.0	1.0	1.0	1.0	1.0
$50,001–100,000	0.90 (0.75–1.09)	1.04 (0.85–1.27)	**1.23** **(1.01–1.51)**	1.02 (0.86–1.23)	**1.43** **(1.20–1.70)**
Over $100,000	1.08 (0.88–1.33)	1.10 (0.88–1.37)	1.19 (0.95–1.48)	1.05 (0.86–1.28)	**1.43** **(1.18–1.72)**
Do not wish to answer	**0.58** **(0.49–0.70)**	**0.75** **(0.61–0.91)**	**0.62** **(0.51–0.74)**	**0.58** **(0.49–0.70)**	**0.76** **(0.64–0.91)**
Patient determined disease steps					
Mild (0–1)	1.0	1.0	1.0	1.0	1.0
Moderate (2–4)	1.16 (0.97–1.39)	1.14 (0.94–1.38)	1.08 (0.89–1.30)	**1.22** **(1.03–1.45)**	1.16 (0.98–1.37)
Severe (5–8)	1.13 (0.91–1.40)	0.92 (0.73–1.16)	1.09 (0.87–1.37)	1.22 (0.99–1.51)	**1.26** **(1.02–1.54)**
Number of comorbidities					
0	1.0	1.0	1.0	1.0	1.0
1	1.07 (0.89–1.30)	1.19 (0.97–1.45)	1.18 (0.96–1.45)	**1.18** **(0.98–1.43)**	1.07 (0.89–1.28)
2	1.07 (0.88–1.31)	**1.28** **(1.04–1.59)**	1.17 (0.94–1.45)	**1.24** **(1.02–1.50)**	1.08 (0.89–1.31)
≥3	1.14 (0.94–1.38)	**1.42** **(1.16–1.75)**	1.04 (0.85–1.28)	**1.37** **(1.14–1.66)**	1.00 (0.84–1.21)
Current smoker	**0.69** **(0.52–0.91)**	0.89 (0.66–1.19)	0.77 (0.58–1.03)	0.91 (0.69–1.20)	0.86 (0.66–1.13)
Any alcohol intake	1.08 (0.94–1.25)	0.96 (0.82–1.12)	1.11 (0.95–1.29)	**1.25** **(1.09–1.44)**	**1.16** **(1.01–1.33)**
Any physical activity	**1.25** **(1.08–1.44)**	1.07 (0.92–1.26)	1.04 (0.89–1.21)	**1.18** **(1.03–1.37)**	1.09 (0.95–1.23)
Religiosity					
5–10	1.0	1.0	1.0	1.0	1.0
11–16	**0.69** **(0.55–0.87)**	0.95 (0.74–1.21)	0.85 (0.66–1.08)	**0.73** **(0.58–0.91)**	0.95 (0.77–1.17)
17–22	**0.63** **(0.49–0.81)**	0.90 (0.69–1.17)	0.77 (0.59–1.00)	**0.63** **(0.50–0.81)**	0.99 (0.78–1.25)
23–27	**0.60** **(0.51–0.70)**	**0.83** **(0.70–0.99)**	**0.80** **(0.67–0.95**)	**0.65** **(0.56–0.76)**	0.99 (0.85–1.15)
Health literacy					
Low	0.62 (0.37–1.05)	0.65 (0.38–1.10)	0.79 (0.46–1.36)	0.72 (0.43–1.21)	0.72 (0.43–1.21)
Marginal	**0.69** **(0.57–0.83)**	0.86 (0.70–1.04)	0.82 (0.68–1.0)	0.89 (0.74–1.07)	0.94 (0.79–1.12)
Functional	1.0	1.0	1.0	1.0	1.0
Clinic course					
Clinically isolated syndrome	1.02 (0.57–1.83)	0.80 (0.45–1.44)	0.83 (0.46–1.50)	0.97 (0.56–1.68)	1.04 (0.60–1.79)
Relapsing remitting	0.84 (0.70–1.01	0.91 (0.75–1.11)	0.87 (0.71–1.05)	0.89 (0.75–1.06)	**0.83** **(0.70–0.99)**
Primary or secondary progressive	1.0	1.0	1.0	1.0	1.0
Don't know	0.85 (0.66–1.09)	0.94 (0.72–1.24)	0.80 (0.61–1.04)	**0.73** **(0.57–0.93)**	**0.76** **(0.60–0.96)**
c-statistic	0.64	0.59	0.61	0.64	0.60
Hosmer-lemeshow goodness of Fit (X^2^)	13.9, *p* = 0.085	4.89, *p* = 0.76	7.31, *p* = 0.50	8.85, *p* = 0.36	3.82, *p* = 0.87
Pseudo, R^2^	0.052	0.0202	0.031	0.057	0.029
Maximum R^2^	0.072	0.030	0.045	0.0770	0.039

Participants evaluated five statements: (i) I would not feel offended if I were asked to participate in a brain donation = NOT OFFENDED; (ii) I am aware of the importance of brain donation for research = AWARE IMPORTANCE; (iii) I believe research staff will handle donated brain samples properly and professionally = TRUST; (iv) I would consider donating my brain for research = CONSIDER DONATION; and (v) I would be agreeable to allow my family to make the decision whether or not to donate my brain for research = ALLOW FAMILY. Bold values indicates statistical significance.

Factors associated with considering brain donation were similar to those associated with not being offended with a few exceptions. Female gender, a higher number of comorbidities and alcohol intake as well as moderate (vs. mild) disability, were also positively associated considering brain donation. Health literacy was not associated with considering brain donation, and older age was slightly negatively associated with considering brain donation as was an unknown clinical course. We did not observe an interaction between clinical course and disability (X^2^ = 8.15, *p* = 0.23).

Factors associated with agreement that the respondent's family could make the decision about brain donation included post-secondary education, higher annual household income, severe disability, and alcohol intake. We did not observe an interaction between clinical course and disability (X^2^ = 10.0, *p* = 0.12).

Factors associated with awareness of the importance of brain donation included female gender, longer disease duration, post-secondary (vs. less than post-secondary) education, and a higher number of comorbidities. The highest (as compared to lowest) degree of religiosity was associated with lack of awareness of the importance of brain donation. We did not observe an interaction between clinical course and disability (X^2^ = 8.58, *p* = 0.20).

Factors associated with trusting that research staff would handle donated brain samples properly and professionally included post-secondary (vs. less than post-secondary) education, and annual income of $50,001–100,000 (vs. < $50,000). We did not observe an interaction between clinical course and disability (X^2^ = 5.35, *p* = 0.50).

### 3.2. Motivations regarding brain donation

The most common reasons for participating in brain donation reported was the potential benefit to other patients in the future, and the advancement of medical knowledge. The most common specific reasons not to participate were the need for more information (22.9%) or time to consider it (21.9%), followed by the potential stress on family members (14.7%). Nearly forty percent of participants indicated “no reasons” (*n* = 1,787).

### 3.3. Communication preferences regarding brain donation

**Figure 2:**
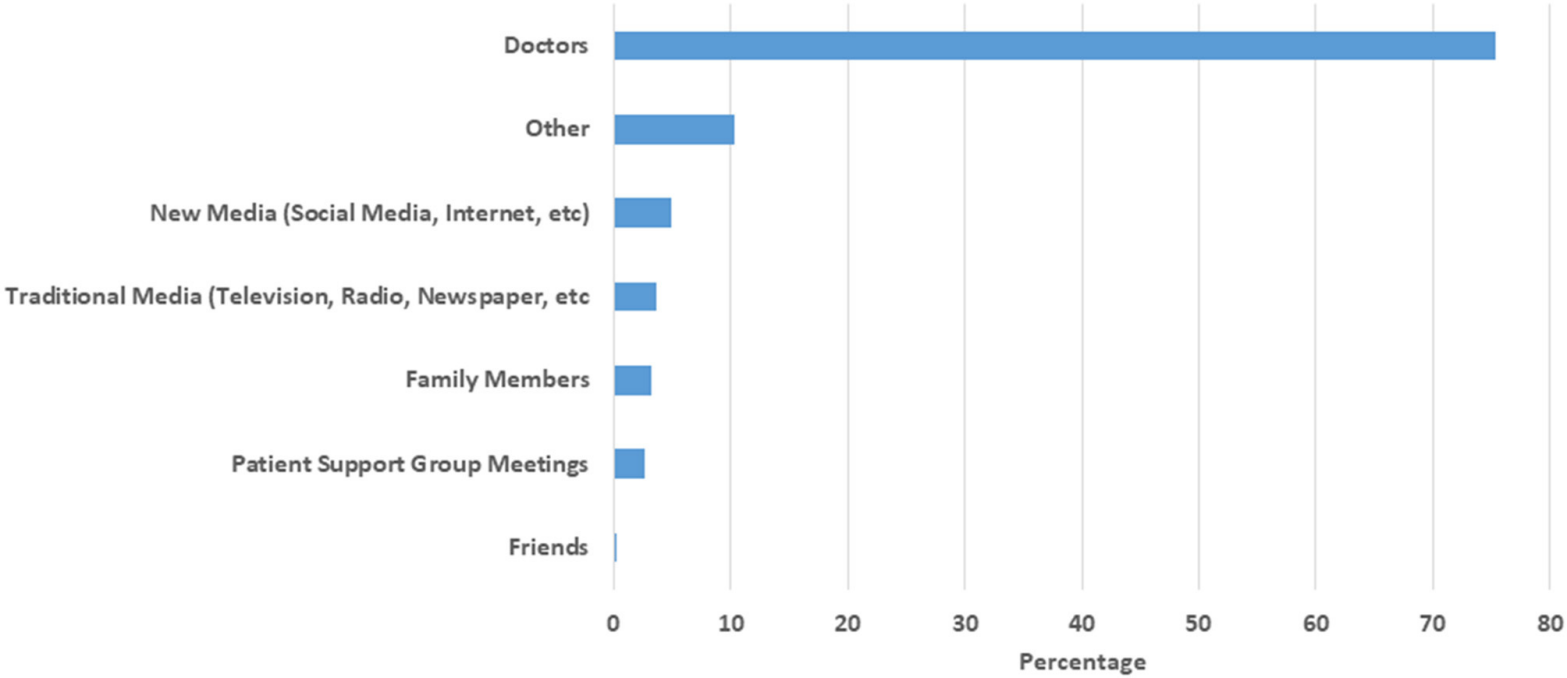
Preferred means of learning about brain donation. Other: Not specified, research group, MS related organizations, religious groups, NARCOMS.

**Figure 3:**
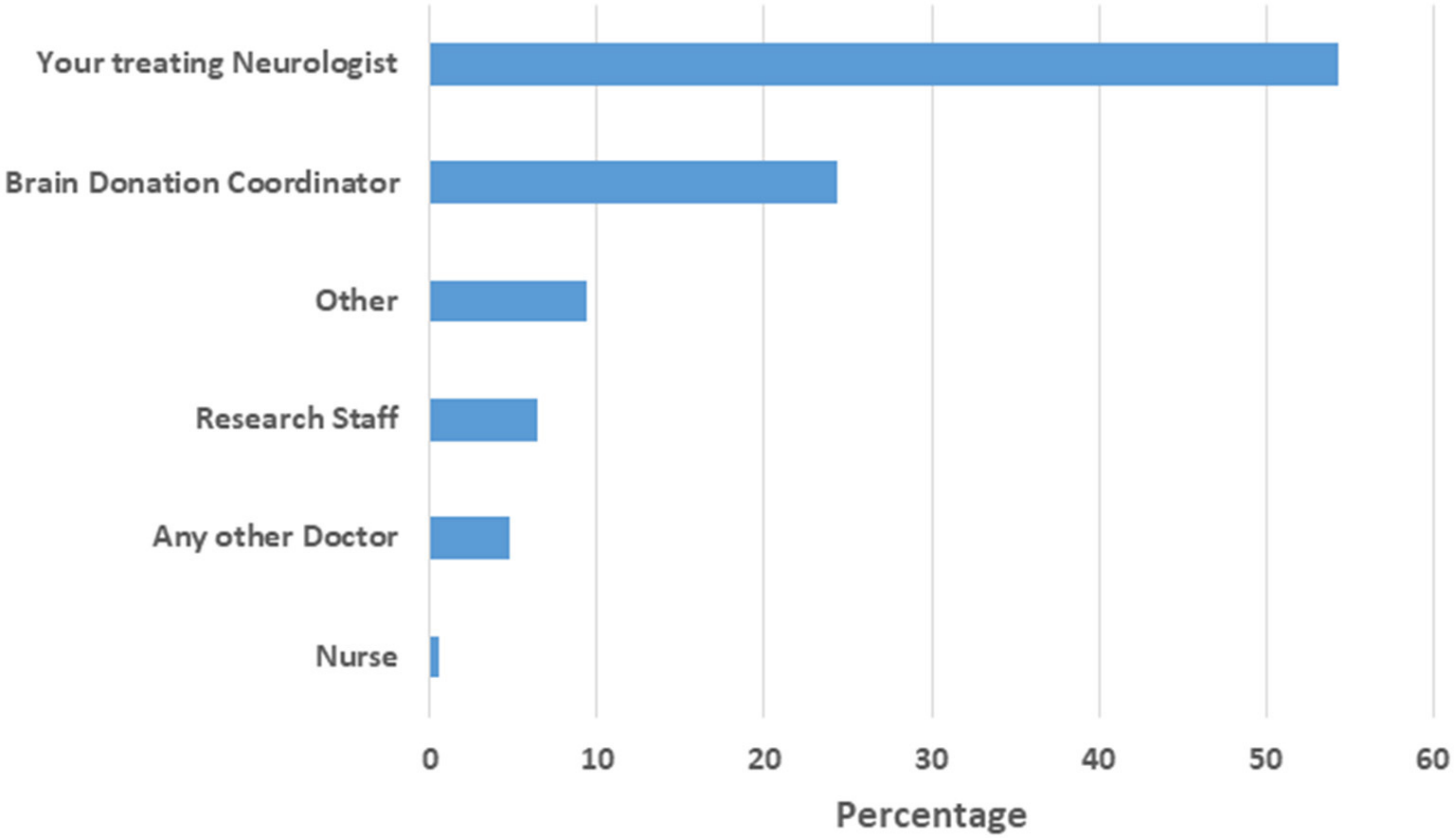
Preferred person to discuss brain donation. Other: family, friend, end of life provider, religious leader, disinterested third party, counselor, unsure.

Of the means of hearing about brain donation, the dominant preference was doctors (Figure 2) with all other means including media, family and friends being preferred by fewer than 5% of participants. The most preferred person to discuss brain donation was the treating neurologist (54.2%), followed by a brain donation coordinator (Figure 3). Nearly half of participants (*n* = 2,179, 48.2%) indicated that they were unsure of the most appropriate time for medical staff to approach them regarding brain donations. Thirty-one percent of participants indicated any time after the first visit was an appropriate time, while 6.3% indicated that the first clinic visit was appropriate, and 7.7% indicated that it would never be appropriate.

## 4. Discussion

In this large cross-sectional study, we investigated attitudes of people with MS toward brain donation, and their preferences related to discussions of brain donation. We found that two out of three participants would consider brain donation. Factors associated with considering brain donation included female gender, having a post-secondary education, being physically active, and a higher burden of comorbidity, as well as any alcohol intake. Health literacy was not associated with considering brain donation, nor was degree of religiosity. However, a higher degree of religiosity and marginal health literacy were associated with disagreement with the statement that they would not be offended if asked to participate in brain donation. Three out of four participants indicated that they preferred to receive information regarding brain donations from physicians, rather than from media sources, support groups, friends or family. One in two participants indicated the preferred person to discuss brain donation with them was their treating neurologist.

We used the same questions as those used in a study of 122 individuals with neurodegenerative disease and 65 individuals with non-neurodegenerative diseases (4). In that study, 68% of those with neurodegenerative disease and 76.9% of those with non-neurodegenerative diseases indicated that they would not be offended if asked to participate in brain donation. This is higher than we observed in a population of comparable age. Findings regarding the proportion indicating they were aware of the importance of brain donation, and trust in research staff were similar for participants with neurodegenerative disease in the prior study and our study. However, participants in our study were more likely to indicate they would consider brain donation (62.2 vs. 48.4–49.2%).

Female gender was associated with willingness to consider brain donation. Findings regarding the association of gender and willingness to donate brain tissue or other organs have been variable depending on the population and type of tissue studied. In the Netherlands, gender was not associated with willingness to participate in brain donation among individuals with psychiatric disorders (11). Among 140 very healthy older individuals in the Oregon Brain Aging Study, gender was not associated with consent rates for brain donation (12). A review noted that women are more likely to become living organ donors than men (13). The author noted that women are generally more altruistic than men (14), but also suggested that women might be more vulnerable to “subtle pressure tapping into more or less conscious role stereotypes.” Females are more likely to report that a decision to participate in a clinical trial is influenced by altruism, as well as by friends, family or researchers than men (15). Further study is needed to understand the gender differences observed herein.

Our findings provide some insight into communication preferences regarding brain donation for people with MS, as well as motivations and barriers. We found that the most common motivations for participating in brain donation were altruistic, whereas lack of information about brain donation, and stress imposed on family members were barriers. An understanding of the rationale and process for brain donation, as well as informed discussions between the patient and trusted health care providers regarding brain donation are important. Among people participating in clinical trials related to Alzheimer's disease, misconceptions related to the utility of studying brain tissue were more common among individuals without a scientific or health care background. Religious beliefs and spirituality also influenced perspectives related to brain donation (16). In contrast, among individuals with movement disorders, willingness to consider brain donation was associated with younger age, but was not associated with gender, religious beliefs or marital status (17). Although religiosity was not associated with willingness to consider brain donation in our sample, individuals with a high degree of religiosity were more likely to indicate they would be offended if asked. The reasons for this are uncertain. A study in Turkey regarding attitudes about organ donation found that a higher score on a religious attitudes (religiosity) scale were associated with fear of medical neglect (18), and concerns about body integrity post-mortem. Lack of knowledge about donation in the context of a particular religion may also be influence attitudes regarding brain donation. A qualitative study of religious immigrants to Sweden found that education about religion, religious aspects of donation, and organization of the health system changed attitudes toward organ donation (19).

Most of our study participants self-identified as white. This limited our ability to identify differences in motivations and barriers across racial and ethnic groups. Communication preferences and health care beliefs may be influenced by cultural factors. Limited work suggests that a lack of trust in health care providers and the health care system by individuals (without MS) who identify as African American or Latino, as well as religious and spiritual concerns are barriers to brain donation (20). This may reflect, in part, historic injustices in research as well as ongoing perceptions and experiences of discrimination when receiving health care (21). Involvement of family members and culturally sensitive information are important to improving brain donation rates in these demographic groups (20), and this topic warrants further investigation in future studies. The Health Equity Through Aging Research and Discussion (HEARD) Study is a mixed methods study that seeks to identify barriers and facilitators of brain donation among older adults who are African American, Latinx or White with lower income (22). A semi-structured interview study found a lack of knowledge regarding brain donation among Latinos, but presentation of information about brain donation lead to substantial support for brain donation to support research on Alzheimer's disease and related dementias (23). Further study of advertising about brain donations (24), the role of educational interventions, and the role of neurologists in delivering those interventions, in improving support for brain donation among the broader population of people with MS is needed. Programs seeking to engage particular populations in brain donation have also pointed to the importance of developing a comprehensive long-term recruitment and retention strategy that involves provision of information, tailored retention activities such as information materials and appreciation events to the participant, engagement of family, among other actions (24, 25). Systematic evaluation of the approaches that are most effective for MS in general may also be valuable.

Our findings should be considered in light of study limitations. The response rate was nearly 67%, slightly exceeding the average response rate of 60% reported in the medical literature (26). Responders differed from non-responders with respect to race, level of education, age and disability status at enrollment but the magnitude of these differences was small. Moreover, NARCOMS participants may not be representative of the entire MS population in the United States, as they predominantly represent older women, of higher socioeconomic status who self-identify as White. The findings also may not generalize to other world regions with differing health systems, religious and cultural beliefs. We did not ascertain specific religions due to sensitivities around this issue in our prior work with this population, but did capture religiosity, a concept that is broader than specific religious beliefs. We lacked information about other characteristics that may influence trust in medical research such as whether the individual had ever worked in a medical setting. Future studies could consider the use of qualitative methods such as focus groups to gain a more detailed understanding of factors influencing attitudes toward brain donation. This could, for example, include assessing prior knowledge and experiences related to tissue donation in general, and exploring the gender differences we observed. Future studies should also explore whether the association of comorbidity with attitudes toward brain donation varies according to the specific comorbidity. Nonetheless, study strengths include the large sample size, and comprehensive assessment of attitudes related to brain donation.

## 5. Conclusion

In summary, two-thirds of people with MS would consider brain donation. Interest in brain donation varies with sociodemographic and disease-related factors and health behaviors, and people with MS desire to hear about brain donation from their health care providers over other sources.

## Data availability statement

The data analyzed in this study were obtained from the North American Research Committee on Multiple Sclerosis Registry (NARCOMS; https://www.narcoms.org/), the following licenses/restrictions apply: Access to these datasets is subject to approval by NARCOMS. Requests to access these datasets should be directed to the NARCOMS Registry, MSregistry@narcoms.org.

## Ethics statement

The studies involving human participants were reviewed and approved by UT Southwestern Institutional Review Board. The patients/participants provided their written informed consent to participate in this study.

## Author contributions

RM: conceptualization, methodology, writing–original draft, writing–review and editing, and supervision. LK: formal analysis and writing–review and editing. GC and RF: conceptualization, methodology, and writing–review and editing. AS: conceptualization, methodology, data curation, and writing–review and editing. All authors contributed to the article and approved the submitted version.
