# Supplementary material for: Attitudes of people with multiple sclerosis toward brain donation
Source: Front Neurol. 2023 Jan 26;14:1115303. doi: 10.3389/fneur.2023.1115303 (PMC9909011; doi:10.3389/fneur.2023.1115303)
Supplement: Supplementary file 1 [file Table_1.DOCX]

Table e1. Characteristics of non-responders and responders

| **Characteristic** | **Non-Responder (N=2849)** | **Responder (N=5719)** | **Std**  **Diff** | **p-value** |
| --- | --- | --- | --- | --- |
| Women, n (%) | 2273 (80.1) | 4624 (80.9) | 0.008 | 0.49 |
| White Race, n (%) | 2375 (84.7) | 4981 (87.5) | 0.03 | <0.001 |
| Education Level, n (%) |  |  |  | <0.001 |
| High school/GED | 795 (31.5) | 1326 (24.7) | 0.15 |  |
| Associate’s Degree | 409 (16.2) | 770 (14.3) | 0.05 |  |
| Bachelor's Degree | 700 (27.8) | 1655 (30.8) | 0.07 |  |
| Post Bachelor’s Degree | 523 (20.8) | 1484 (27.6) | 0.16 |  |
| Technical Degree | 93 (3.7) | 136 (2.5) | 0.07 |  |
| Age at Symptom Onset (years), n (%) | 30.7±10.9 | 31.2±10.5 | 0.05 | 0.065^a^ |
| Year Enrolled | 2007.9±7.7 | 2005.5±6.6 | 0.34 | <0.001^a^ |
| Age at Enrollment (years), mean (SD) | 48.1±10.4 | 47.3±9.8 | 0.08 | <0.001^a^ |
| PDDS at enrollment, n (%) |  |  |  | ***<0.001^c^*** |
| Mild (0-1) | 842 (29.6) | 2150 (37.6) | 0.17 |  |
| Moderate (2-4) | 1073 (37.7) | 788 (13.8) | 0.57 |  |
| Severe (5-8) | 667 (23.4) | 920 (16.1) | 0.18 |  |

Std diff = standardized difference. Small = 0.2-0.49, Medium = 0.50-0.79, Large ≥ 0.80
